# Human papillomavirus genotypes and P16INK4A expression in squamous penile carcinoma in Mexican patients

**DOI:** 10.1186/s12879-019-4696-6

**Published:** 2019-12-19

**Authors:** Cecilia Martínez-Bailón, Alejandra Mantilla-Morales, Galo Méndez-Matías, Isabel Alvarado-Cabrero, Rogelio Maldonado-Rodríguez, Joel Quintero-Becerra, Rafael Arias-Flores, Patricia Piña-Sánchez

**Affiliations:** 1 Molecular Oncology Laboratory, Oncology Research Unit, Instituto Mexicano del Seguro Social, CMN S XXI IMSS, Mexico City, Mexico; 2 Department of Pathology, UMAE Oncology Hospital, Instituto Mexicano del Seguro Social, CMN S XXI IMSS, Mexico City, Mexico; 3 Department of Biochemistry, Escuela Nacional de Ciencias Biológicas, Instituto Politécnico Nacional, Mexico City, Mexico; 4 Department of Urology, UMAE Oncology Hospital, Instituto Mexicano del Seguro Social, CMN S XXI, Mexico City, Mexico; 5 Department of Epidemiology, UMAE Pediatrics Hospital, Instituto Mexicano del Seguro Social, CMN S XXI, Mexico City, Mexico

**Keywords:** Penile carcinoma, Mexico, HPV, P16INK4A, Multiple genotypes

## Abstract

**Background:**

Approximately 50% of cases of penile carcinoma (PeCa), a rare neoplasm worldwide, are associated with human papillomavirus (HPV). However, the detection of HPV-DNA is not sufficient to consider it the etiological factor in the development of this type of cancer. Currently, the overexpression of P16INK4A is used as a surrogate biomarker of HPV carcinogenesis. Information on PeCa in Mexico is scarce, particularly regarding cases related to HPV and genotype frequency.

**Objective:**

To evaluate the presence of HPV, its genotypes, and the presence of multiple genotypes, and the expression of P16INK4A, as well as its clinical and histopathological parameters.

**Methods:**

For HPV-DNA detection and P16INK4A expression, we used the INNO-LiPA® test and immunohistochemistry, respectively.

**Results:**

Sixty cases of PeCa were evaluated, of which 75% were HPV-non-related histological variants. We found that 58.9% (33/56) of PeCa cases were HPV-DNA positive, while 30.9% of the cases evaluated (17/55) were positive for P16INK4A. HPV16 was the main genotype in 42.9% of the cases, followed by HPV52 in 7.1% and HPV18 in 5.4%. Within the HPV-positive cases, 27.3% had multiple genotypes. All HPV-positive patients under the age of 45 years were positive only for HPV16.

**Conclusions:**

HPV16 was the most commonly detected genotype in PeCa. HPV 31, 35 and 39 were infrequent; however, they were related to a single infection and P16INK4A overexpression; thus, they seem to be relevant in PeCa carcinogenesis. Our results suggest that P16INK4A overexpression could be useful for the classification of HPV-related PeCa. The role of multiple HPV genotypes in the development and prognosis of PeCa is still not completely understood. Thus, it is necessary to define criteria to establish reliable ways to classify HPV-related PeCa that could lead to optimal therapeutic approaches.

## Background

Penile carcinoma (PeCa) is a globally rare neoplasia that mainly affects men between 50 and 70 years of age. The incidence reported in 2012 was 26,000 new cases worldwide [1]. PeCa is most frequent in developing countries, particularly in regions of Africa, South America and Asia, where PeCa may represent up to 10% of all tumors in men [2].

Ninety-five percent of all PeCa cases are of the squamous type, originating in the internal mucosa of the glans, coronal sulcus or skin. The current histopathological classification from the WHO regarding the human papillomavirus (HPV) states that among the histological subtypes of squamous cell carcinoma not related to HPV are usual, pseudohyperplastic and pseudoglandular, verrucous, papillary and adenosquamous. Other types not associated with HPV are sarcomatoid and mixed [3, 4]. The risk factors involved in these subtypes are chronic inflammation, phimosis, lichen sclerosus and lichen planus [5].

Between 30 and 50% of all cases of invasive PeCa are related to HPV, and the main histological subtypes are basaloid and warty. Warty carcinoma includes classical, clear cell carcinoma and warty-basaloid. The basaloid type can be subdivided into the classic and papillary subtypes. Basaloid carcinomas have a high rate of regional metastasis, while warty carcinomas are rarely metastatic. Other less frequent tumors are lymphoepithelioma and medullary squamous cell carcinoma [1, 3].

HPV16 is the most frequent genotype in PeCa throughout the world, regardless of geographic region. However, the order of frequency of other genotypes varies with region. For example, HPV genotypes 30, 33 and 52 are more present in Africa; 6, 11 and 33 in America; 33, 35 and 45 in Asia; and 52, 6 and 33 in Europe. It is interesting to note that low-risk genotypes 6 and 11 are regularly detected in PeCa. In fact, a recent meta-analysis reported that HPV16 was the second most common genotype in PeCa and the third most common genotype as a single infection after HPV18 [6–8].

Current studies have emphasized that the detection of viral DNA is not sufficient to establish a causal relationship between HPV and cancer. Thus, other surrogate biomarkers of transformation induced by HPV infection, such as overexpression of P16INK4A, should also be evaluated, as well as the presence of viral activity by detection of expression of viral oncoproteins, such as E6*I [9, 10].

Next-generation sequencing has shown a broad spectrum of HPV in both mucosal and skin epithelium, where a wide variety of genotypes of alpha, beta and gamma genera have been identified [11]. Nevertheless, the IARC only includes 12 genotypes as type 1 carcinogens at present, and the oncogenic capacity of other genotypes is currently being assessed according to epidemiology and molecular alterations that are characteristic of HPV-induced carcinogenesis [10].

In Mexico, there are several reports on the frequency and diversity of HPV, mainly in cervical cancer [12–14]. However, studies on PeCa and HPV are scarce. López et al. identified HPV in 78% of PeCa cases using GP primers, finding that the most prevalent genotypes were HPV 16, 31, and 11 [15]. Other works have addressed the presence of HPV in penile samples from the general population, where HPV was identified in 62% of the cases [16]. In a study analyzing penile intraepithelial lesions, HPV was detected in all cases, 80% of which were positive for multiple genotypes [17]. To contribute to the knowledge of the etiopathogenesis of PeCa in Mexico, we evaluated a group of 60 patients with a diagnosis of PeCa and determined the frequency of HPV, the genotypes of HPV, and the presence of multiple HPV genotypes, and expression of P16INK4A as a surrogate marker, as well as the clinical and histopathological characteristics, of PeCa according to the WHO 2016 classification.

## Methods

### Patients

Sixty cases of penile carcinoma were collected from pathology archives of the Oncology Hospital, National Medical Center S XXI, Instituto Mexicano del Seguro Social (IMSS). The project was approved by The Research and Ethics Committee (R-2014-3602-26). An expert pathologist revised the histopathological description of each case according to the 2016 WHO classification of penile cancer [4]. Tissues were selected for tissue microarray (TMA) construction and DNA extraction.

### HPV genotyping

Paraffin-embedded tissues were sectioned for DNA extraction using the Wizard FFPE Kit from a ReliaPrep™ FFPE gDNA Miniprep System Wizard (Promega) kit. HPV detection was carried out using an INNO-LiPA® HPV Genotyping Extra Test (Innogenetics now Fujirebio) commercial kit. This kit allows the simultaneous detection of 28 viral types classified according to the IARC as follows: Group I: HPV16, 18, 31, 33, 35, 39, 45, 51, 52, 56, 58, 59; Group 2A: VPH68; Group 2B: HPV26, 53, 66, 69, 70, 73, 82; Group 3: HPV6 and 11; and others: HPV 40, 43, 44, 54, 71, 74. The technique is based on PCR amplification with SPF10 primers and reverse line hybridization with specific probes for the mentioned genotypes. The process was performed on an Autolipa™ 48 device, and the results were interpreted by Liras® software for LIPA HPV V2. Only samples with a positive reaction in the HLA-DPB1 control were included in the analysis. Any genotype not clearly present according to the results issued by the software was not considered in the analysis.

### Tissue microarray and P16INK4A immunohistochemistry

Representative tumor areas were selected for the construction of a tissue microarray (TMA) with an Advance Tissue Array™ ATA-100 instrument (Chemicon International). Two or three 1 mm diameter tissue cores from each of the samples were placed in a receptor paraffin block. Control tissues from cervical cancer and tonsil tissue were included in the TMA. Histological, 4 μm thick sections were mounted on adhesive slides. The detection of P16INK4A was performed by immunohistochemistry with a CINtec® histology antibody (E6H4 clone) in an automated Benchmark Autostainer™ device (Ventana Medical Systems Inc.). The reaction was developed using an UltraView™ Universal DAB kit (Ventana Medical Systems Inc.). Tissues were stained with hematoxylin and dehydrated and mounted with synthetic resin.

Positivity for P16INK4A was considered when more than 70% of the cells presented a strong and diffuse, nuclear and/or cytoplasmic staining pattern, according to the Larsen study [18]. For those cases that were HPV-DNA positive and P16INK4A-negative in TMA, a new immunohistochemical test was performed in the complete tissue block.

### Statistical analysis

Data were processed with the PASW Statistics™ version 18.0 (IBM Corporation, Somers NY) statistical package. Qualitative variables were described as frequencies and percentages. The association between clinicopathological variables was evaluated using the chi-squared test and Fisher’s exact test. A logistic regression model was used to obtain the contribution of HPV genotypes and morphological classification. Values of p below 0.05 were considered statistically significant.

## Results

**Fig. 1:**
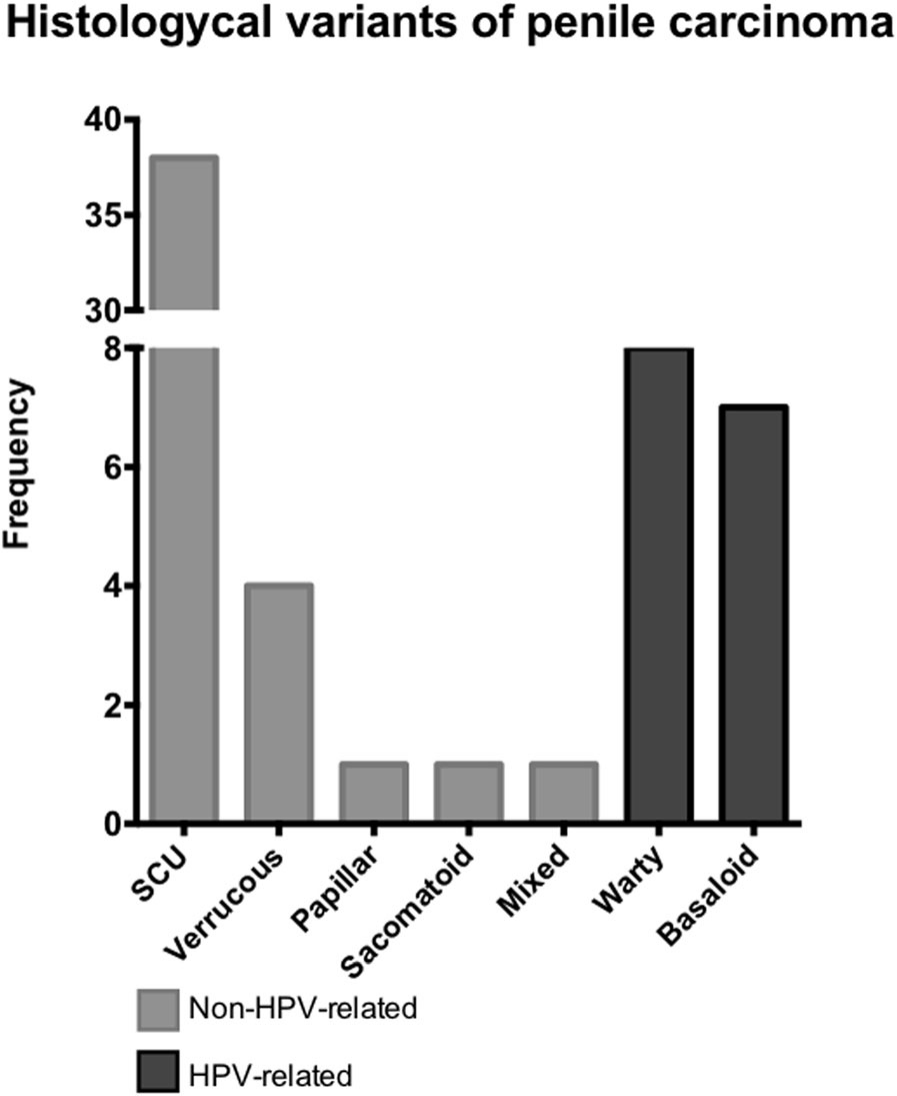
Histology classification of penile squamous cell carcinoma. The classification was done according to 2016 WHO

Sixty cases diagnosed as squamous cell carcinoma of the penis were obtained from pathology archives. Patients ranged from 24 to 95 years of age, with a mean of 62.3 years ±16.4. Patients below the age of 45 years comprised 13.3% of the total. The most common anatomic site of origin was the glans in 59% of the cases, while 20% of the cases involved two or more origin sites (multifocal). Primary treatment performed on 70% of the patients was total penectomy, while the remaining 30% underwent a partial procedure. Regarding the size of the tumors, 63.5% were larger than 5 cm at the time of diagnosis. Clinical stages I and II were present in 34.2% of the patients, and stages III and IV were present in 65.7% (Table 1). Regarding histological types, 75% of the cases were non-HPV related; among them, the most common variant was squamous cell carcinoma of the usual type (Fig. 1, Table 2).

**Table 1:** Descriptive clinical characteristics of penile cancer

Characteristics	Total		HPV Positive		HPV MG		P16INK4A+	
	*n* = 60	%	*n* = 56	55%	n = 33	%	*n* = 55	30.9%
Age diagnosis								
≤ 45	8	13.3	4/7	57.1	0	0.0	2/6	33.3
46–55	13	21.7	5/12	41.7	1	20.0	0/12	0.0
56–65	11	18.3	5/10	50.0	3	60.0	3/10	30.0
66–75	12	20.0	8/12	66.7	1	12.5	4/12	33.3
≥ 76	14	23.0	10/13	76.9	5	50.0	7/13	53.8
¿?	2	3.3	1/2	50.0	0	0.0	1/2	50.0
Tobacco								
Yes	16	37.2	7/15	46.7	4	57.1	3/15	20.0
No	27	62.8	16/25	64.0	5	31.3	8/24	33.3
Alcohol								
Yes	10	23.3	5/9	55.6	5	100.0	3/9	33.3
No	33	76.7	18/31	58.1	4	22.2	8/30	26.7
					*p* = 0.0037			
Anatomical region								
Corpus	2	4.1	1/2	50.0	1	100.0	1/2	50.0
Glans	29	59.2	15/7	55.6	3	20.0	4/6	15.4
Prepuce	6	12.2	2/6	33.3	2	100.0	2/6	33.3
Coronal surcus	2	4.1	2/2	100.0	0	0.0	1/2	50.0
Multifocal	10	20.4	7/10	70.0	3	42.9	5/10	50.0
Tumoral size								
< 5 cm	15	36.6	10/15	66.7	4	40.0	5/15	33.3
5–10 cm	17	41.5	10/16	62.5	1	10.0	5/15	33.3
> 10 cm	9	22.0	4/7	57.1	4	100.0	2/8	25.0
Clinical Stage								
I	6	17.1	5/6	83.3	2	40.0	1/5	20.0
II	6	17.1	3/4	75.0	1	33.3	2/5	40.0
III	11	31.4	7/11	63.6	2	28.6	3/10	30.0
IV	12	34.3	5/11	45.5	1	20.0	2/11	18.2

*HPV MG* HPV Multiple Genotypes

**Table 2:** HPV presence and P16INK4A expression in penile squamous cell carcinomas

	Total		HPV-		HPV+		HPV MG		P6INK4A+		P16INK4A-	
	n	%	n	%	n	%	n	%	n	%	n	%
HPV-non-related carcinoma	45	75.0	17	41.5	24	58.5	5	20.8	11	27.5	29	72.5
Squamous cell carcinoma usual	38	63.3	15	42.9	20	57.1	4	20.0	11	32.4	23	67.6
Verrucous	4	6.7	1	25.0	3	75.0	0	0.0	0	0.0	4	100.0
Papillary NOS	1	1.7	1	100.0	0	0.0	0	0.0	0	0.0	1	100.0
Sarcomatoid	1	1.7		N.D.		N.D.		N.D.		N.D.		N.D.
Mixed	1	1.7	0	0.0	1	100.0	1	100.0	0	0.0	1	100.0
HPV-related penile carcinoma	15	25.0	6	40.0	9	60.0	5	55.6	6	40.0	9	60.0
Basaloid	7	11.3	2	28.6	5	71.4	3	60.0	5	71.4	2	28.6
Warty	8	13.3	4	50.0	4	50.0	2	50.0	1	12.5	7	87.5
Histologycal grade												
Grade 1	27	45.0	16	61.5	10	38.5	3	30.0	3	12.5	21	87.5
Grade 2	25	41.7	7	30.4	16	69.6	4	25.0	9	37.5	15	62.5
Grade 3	8	3.3	0	0.0	7	100.0	3	42.9	5	71.4	2	28.6
					*p* = 0.0053				*p* = 0.0079			

The histological classification was carried out according to the WHO 2016 criteria. The HPV detection was performed using Inno-Lipa, only 56 cases were valid. Regarding P16INK4A, the evaluation was performed in 55 cases. Statistically significant differences are indicated *p* < 0.05 (chi square test)

*HPV MG* HPV multiple genotypes, *N.D* Not determinate

**Fig. 2:**
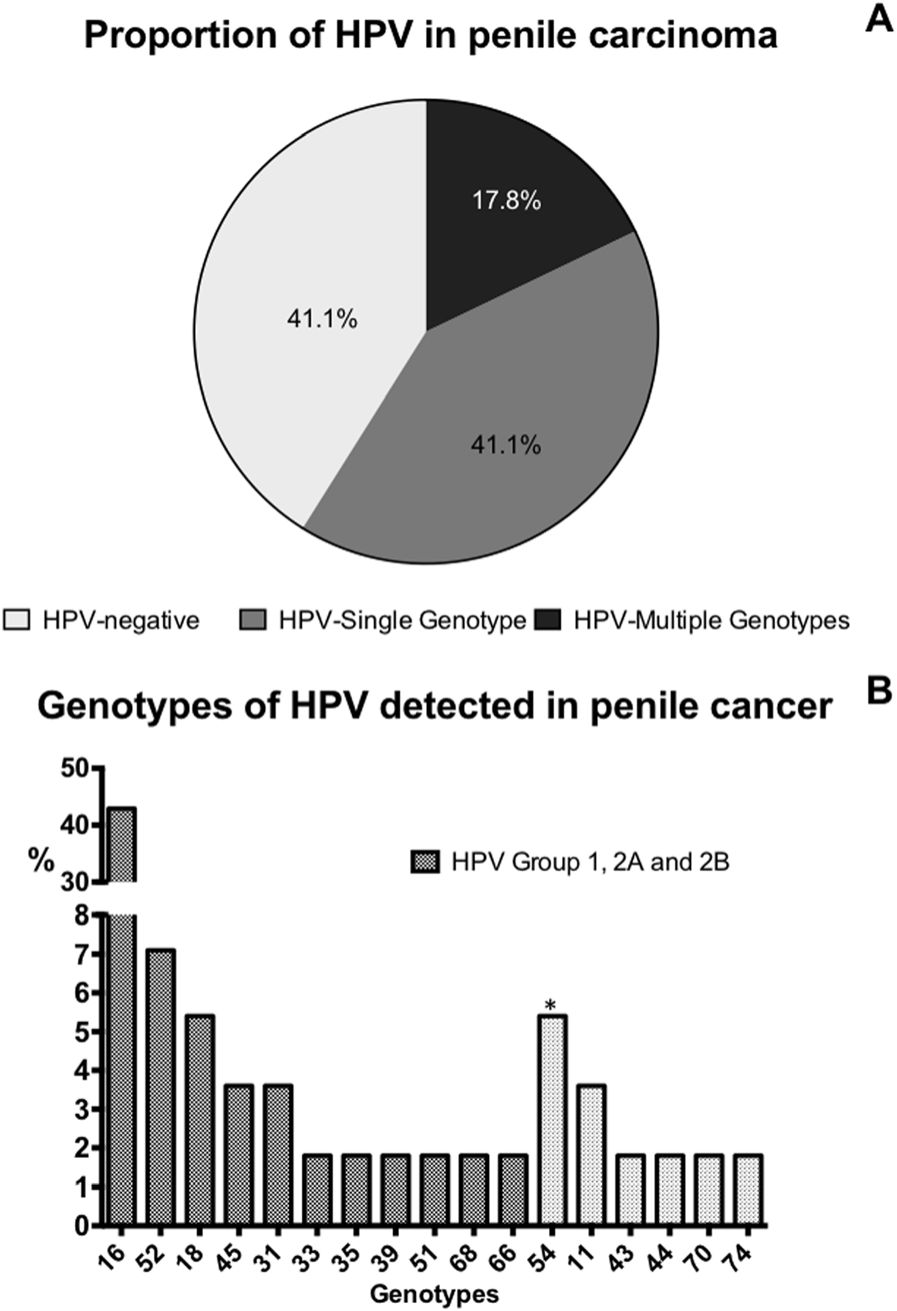
Human papillomavirus DNA in penile carcinoma. **a** proportion of HPV negative cases, HPV single genotype, and HPV multiple genotypes. **b** The frequency of HPV genotypes and carcinogenic classification according to the IARC. * The possible presence of HPV is not ruled out

Four cases were excluded because the control gene was not amplified, leaving 56 cases. Of these, 58.9% (*n* = 33) were HPV positive, of which 69.7% (*n* = 23) had only one viral genotype (single genotype) and 30.3% (*n* = 10) had more than one viral type (multiple genotypes) (Fig. 2a). Among the sixteen different viral genotypes detected, HPV16 had the highest frequency (42.9%) (Fig. 2b), followed by HPV52 (7.1%) and HPV18 (5.4%). The most frequent low-risk genotype was HPV11 (3.6%). These data reflect only the genotypes accurately identified by the test [19]. The WHO morphological classification (HPV-related) was not associated with HPV-DNA molecular detection (*p* = 0.9).

Nevertheless, because of the detection method used (INNO-LiPA®), the presence of HPV52 and HPV54 with other high-risk genotypes, such as 16, 31 and 33, could not be ruled out, as well as HPV 39 with HPV 18. Of the HPV16 positive cases, 15 were identified in single infections, and 7 were identified with other genotypes, such as HPV 18, 66, 33, 45 and 52. It is important to note that HPV18 was only found together with other high-risk genotypes, such as HPV 16, 45 and 51.

**Fig. 3:**
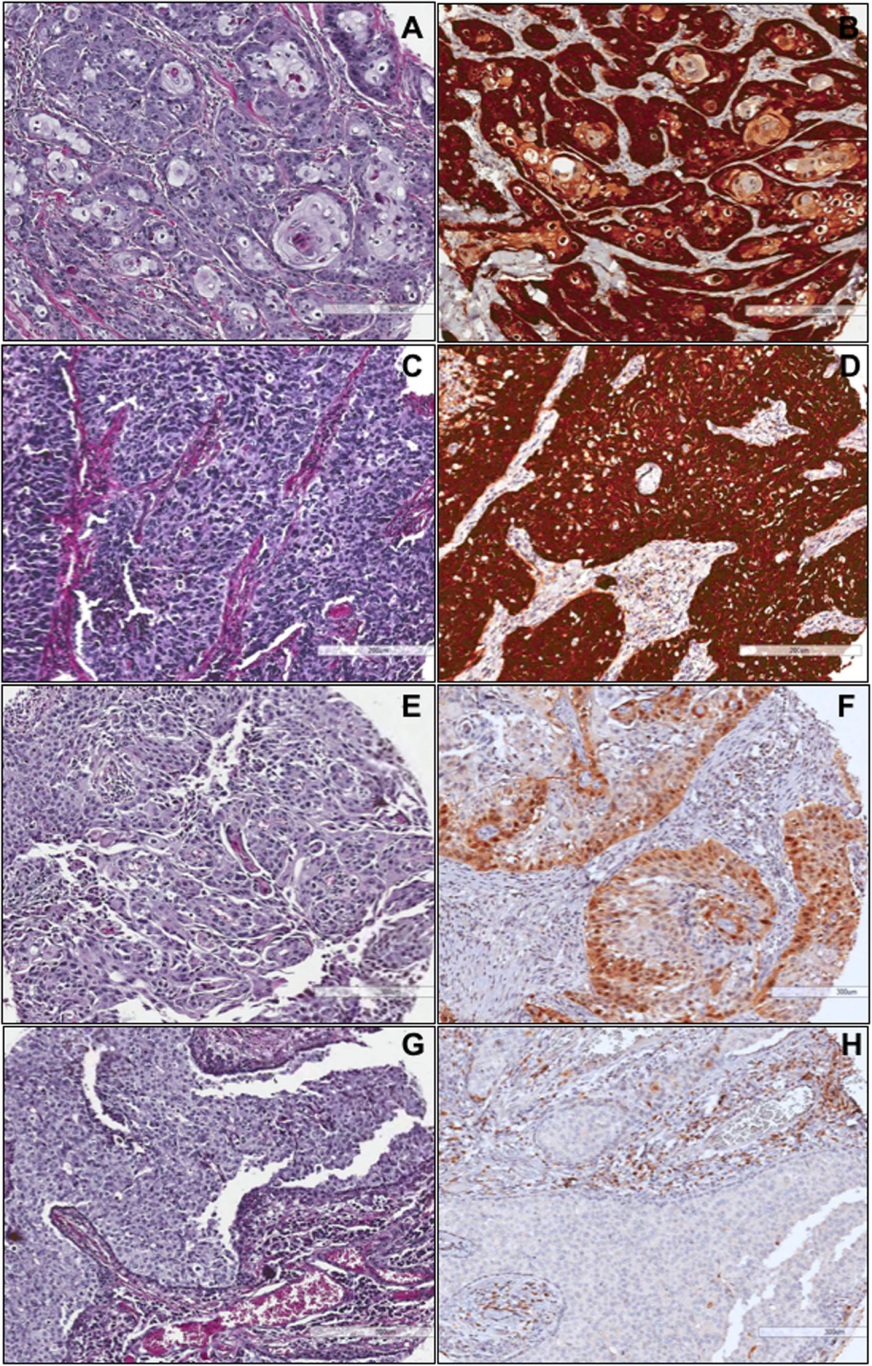
Expression of P16INK4A in penile carcinoma. **a** and **b** correspond to an epidermoid case of the usual type moderately differentiated with HPV 31 and positive to P16INKA. **c** and **d**, poorly differentiated basaloid variant epidermoid carcinoma, positive for HPV35 and P16INK4A. **e** and **f**, usual moderately differentiated carcinoma negative to HPV with low expression of P16INK4A. **g** and **h**, basaloid carcinoma moderately differentiated negative to HPV and P16INK4A

The presence of P16INK4A was analyzed in 55 samples. Of these, 17 (30.9%) were considered positive, as they presented a pattern of intense, diffuse staining in more than 70% of the tissue analyzed (Fig. 3). All samples positive for P16INK4A also presented with HR-HPV. However, of all the samples positive for HR-HPV, only 60.7% were positive for P16INK4A (11 cases positive for HR-HPV were negative for P16INK4A). In these cases, the immunohistochemical test was repeated in complete tissue blocks. All of them were negative (not shown). Regarding histological characteristics, the expression of P16INK4A and the presence of HPV were significantly associated with the degree of differentiation (*p* < 0.05) (Table 1).

## Discussion

In the present study of 60 cases of squamous PeCa, although 45 (75%) were histologically classified as non-HPV-related, 33 of 56 cases tested (58.9%) were positive for HPV-DNA. A recent meta-analysis showed that, on average, 50.8% of PeCa cases presented with HPV [8]. According to ICO reports, the frequency of HPV-DNA in PeCa oscillates between 87% in South Africa and 13.3% in Asia [6], indicating differences with respect to geographical regions. Interestingly, our work is in contrast with a previous report from Mexico, in which 78% of cases were identified as positive by using GP5/GP6+ primers [15]. It is noteworthy that, in comparison, the INNO-LiPA® method is considered a more sensitive technique [20].

In the WHO classification of PeCa, the “usual type” variant is categorized as not related to HPV [3, 4]. However, we did not find differences between the presence of HPV-DNA and the histological classification of “HPV-related”. Because in many cases only one representative block was evaluated, it is possible that the mixed variant may not have been identified in some cases. In this study, all cancers with P16INK4A overexpression had HPV-DNA detected. However, not all cases positive for HPV-DNA were positive for P16INK4A. Some evidence suggests that the detection of viral DNA is not sufficient for the etiological attribution of HPV-related cance; but is necessary to identify transcriptional activity or molecular signature of HPV-induced carcinogenesis [9, 10].

Worldwide, the most frequent genotypes reported in PeCa are HPV16, 6, 33, 35, 45 and 52 [6]. In our work, we identified the following genotypes in descending order: 16, 52, 18, 45, 31 and 11. The distribution of high-risk genotypes different from HPV16 varies with geography and anatomical site [6, 21]. According to several reports, HPV18 is not found within the five most frequent genotypes in PeCa, either globally or by continent [6]. In fact, in some studies, such as the one by de Andrade et al., HPV18 was not detected at all [22]. The differences may be because HPV18 preferentially infects and transforms columnar epithelial tissue, such as in cervical adenocarcinomas [23]. Interestingly, Senba et al. reported HPV18 as the most common genotype in PeCa in Thailand (55%) [24]. A high frequency of HPV18 in cervical cancer, particularly in adenocarcinoma (53%), has also been reported in Thailand [25]. In the present study, HPV18 was the third most frequent genotype, although we only found it together with other high-risk genotypes.

The presence of multiple HPV genotypes has been explored mainly in cervical cancer and its precursor lesions [14, 26–28]. Some studies have suggested an association between an increase in the risk of cervical disease and the cumulative number of HPV genotypes [28], whereas other studies have reported that the number of genotypes present has no additive effect on the risk of precursor lesions [26]. To date, only a few studies have analyzed the presence of multiple HPV genotypes in PeCa. Fernandez et al. [29] recently reported that 53% of penile intraepithelial neoplasia (PeIN) presented multiple HPV genotypes. Rantshabeng et al. reported a higher frequency of multiple HPV genotypes in HIV-positive patients compared to HIV-negative patients with anogenital cancer, including penile cancer [30]. A high prevalence of multiple HPV genotypes (59%) has also been reported in the exfoliative cytology (urethra, penis, scrotum anus) of men referred for evaluation of genital lesions such as warts and sexually transmitted infections, finding up to 9 viral genotypes [31]. Afonso et al. identified multiple genotypes in 23% of HPV-positive samples of PeCa, where HPV16 was found in almost half of the samples [32]. In our study, we identified a similar proportion of multiple genotypes (30%) in HPV-positive cases. To date, however, the role of multiple genotypes in the development and progression of PeCa has not been addressed in detail.

Regarding the patient’s age, we found that 57% of patients under 45 years were HPV positive, and all of them had a single HPV16 infection. In contrast, 76% of older patients (> 76 years) were HPV positive, and only one half of them presented a single genotype (HPV16 or HPV35). These data suggest a relationship between genotype and age, possibly due to fast progression caused by a variety of events such as chromosomic instability and viral integration associated with the oncogenic capacities of E6 and E7 [33–36]. Although one of the limitations in the present work is the number of samples analyzed, the data show a tendency that associates age with the presence of multiple genotypes. Patient grade 3 differentiation shows an association with HPV-positive tumors, in concordance with a previous report [37].

Although P16INK4A has been defined as a surrogate marker for high-risk HPV-induced transformation, differences have been observed in the concordance between HPV presence and P16INK4A expression [29, 38]. Some studies have reported approximately 10% of PeIN cases that are HR-HPV positive and negative for P16INK4A [29] and up to 50% of cases in PeCa [22]. Some explanations attribute this to a transitory infection of HPV, unrelated to the carcinogenic process. Another cause may be the epigenetic mechanisms of the inactivation of *CDNK2A*, as well as loss of heterozygosity [39]. Therefore, the detection of viral transcripts and surrogated markers can be used to define cases attributed to HR-HPV [40].

## Conclusions

In our study, HPV was detected in almost 60% of PeCa cases, although three quarters were classified as non-HPV-related tumors. HPV16 was found to be the most frequent genotype present, followed by HPV52, 33, 35, and 39. Most of these could be prevented by nonavalent HPV vaccination. The role of multiple HPV genotypes in PeCa is of interest. The etiological attribution to HPV in PeCa may be stratified with more certainty by using biomarkers such as viral oncoprotein mRNA and surrogate markers such as P16INK4A.

## Data Availability

The datasets used and/or analyzed during the current study are available from the corresponding author on reasonable request.

## Abbreviations

A.C.: Civil Association
CMN S XXI: Centro Médico Nacional Siglo XXI
DNA: Deoxy-ribonucleic Acid
FFPE: Formalin Fixed Paraffin Embedded
HPV: Human Papillomavirus
HR-HPV: High-Risk Human Papilloma Virus
IARC: International Agency for Research on Cancer
IMSS: Instituto Mexicano del Seguro Social
MG: Multiple Genotypes
PeCa: Penile Carcinoma
WHO: World Health Organization

## References

[1] Plummer M, de Martel C, Vignat J, Ferlay J, Bray F, Franceschi S (2016). Global burden of cancers attributable to infections in 2012: a synthetic analysis. Lancet Glob Health.

[2] Bleeker MCG, Heideman DAM, Snijders PJF, Horenblas S, Dillner J, Meijer CJLM (2009). Penile cancer: epidemiology, pathogenesis and prevention. World J Urol.

[3] Moch H, Cubilla AL, Humphrey PA, Reuter VE, Ulbright TM (2016). The 2016 WHO classification of Tumours of the urinary system and male genital organs-part a: renal, penile, and testicular tumours. Eur Urol.

[4] Cubilla AL, Velazquez E, Amin M, Epstein J, Berney D, Corbishley C (2018). The World Health Organization 2016 classification of penile carcinomas: a review and update from the International Society of Urological Pathology expert driven recommendations. Histopathology..

[5] Ottenhof SR, Bleeker MCG, Heideman DAM, Snijders PJF, Meijer CJLM, Horenblas S, Muneer A, Horenblas S (2016). Etiology of penile cancer. Textbook of penile cancer.

[6] Bruni L, Barrionuevo-Rosas L, Serrano B, Mena G, Muñoz J, Bosch F (2017). Human papillomavirus and related diseases report WORLD. ICO Inf Cent HPV Cancer.

[7] Alemany L, Cubilla A, Halec G, Kasamatsu E, Quirós B, Masferrer E (2016). Role of human papillomavirus in penile carcinomas worldwide. Eur Urol.

[8] Olesen TB, Sand FL, Rasmussen CL, Albieri V, Toft BG, Norrild B (2019). Prevalence of human papillomavirus DNA and p16INK4a in penile cancer and penile intraepithelial neoplasia: a systematic review and meta-analysis. Lancet Oncol.

[9] Halec G, Schmitt M, Dondog B, Sharkhuu E, Wentzensen N, Gheit T (2013). Biological activity of probable/possible high-risk human papillomavirus types in cervical cancer. Int J Cancer.

[10] Arbyn M, Tommasino M, Depuydt C, Dillner J (2014). Are 20 human papillomavirus types causing cervical cancer?. J Pathol.

[11] Brancaccio RN, Robitaille A, Dutta S, Cuenin C, Santare D, Skenders G (2018). Generation of a novel next-generation sequencing-based method for the isolation of new human papillomavirus types. Virology..

[12] Piña-Sánchez P, Hernández-Hernández DM, López-Romero R, Vázquez-Ortíz G, Pérez-Plasencia C, Lizano-Soberón M (2006). Human papillomavirus-specific viral types are common in Mexican women affected by cervical lesions. Int J Gynecol Cancer.

[13] Salcedo M, Piña-Sánchez P, Vallejo-Ruiz V, Monroy-Garcia A, Aguilar-Lemarroy A, Cortes-Gutierrez EI (2014). Human papillomavirus genotypes among females in Mexico: a study from the Mexican institute for social security. Asian Pac J Cancer Prev.

[14] Aguilar-Lemarroy A, Vallejo-Ruiz V, Cortés-Gutiérrez V, Salgado-Bernabe M, Ramos-González N, Ortega-Cervantes L (2015). Human papillomavirus infections in Mexican women with Normal cytology, precancerous lesions, and cervical Cancer: type-specific prevalence and HPV Coinfections. J Med Virol.

[15] Lopez-Romero R, Iglesias-Chiesa C, Alatorre B, Vazquez K, Piña-Sanchez P, Alvarado I (2013). HPV frequency in penile carcinoma of Mexican patients: important contribution of HPV16 european variant. Int J Clin Exp Pathol.

[16] Giuliano AR, Lazcano-Ponce E, Villa LL, Flores R, Lee J, Papenfuss MR (2008). The human papillomavirus infection in men study: human papillomavirus prevalence and type distribution among men residing in Brazil, Mexico, and the United States. Cancer Epidemiol Biomark Prev.

[17] Sudenga SL, Torres BN, Fulp WJ, Silva R, Villa LL, Lazcano-Ponce E (2017). Country-specific HPV-related genital disease among men residing in Brazil, Mexico and the United States: the HIM study. Int J Cancer.

[18] Larsen CG, Gyldenløve M, Jensen DH, Therkildsen MH, Kiss K, Norrild B (2014). Correlation between human papillomavirus and p16 overexpression in oropharyngeal tumours: a systematic review. Br J Cancer.

[19] Innogenetics (2011). INNO-LiPA HPV genotyping extra amp.

[20] Kleter B, Van Doorn LJ, Ter Schegget J, Schrauwen L, Van Krimpen K, Burger M (1998). Novel short-fragment PCR assay for highly sensitive broad-spectrum detection of anogenital human papillomaviruses. Am J Pathol.

[21] Gillison ML, Castellsagué X, Chaturvedi A, Goodman MT, Snijders P, Tommasino M (2013). Eurogin roadmap: comparative epidemiology of HPV infection and associated cancers of the head and neck and cervix. Int J Cancer.

[22] De Andrade MV, Pinho JD, Júnior AALT, Nogueira LR, Silva FF, Maulen VE (2018). P16INK4a expression in patients with penile cancer. PLoS One.

[23] Bulk S, Berkhof J, Bulkmans NWJ, Zielinski GD, Rozendaal L, Van Kemenade FJ (2006). Preferential risk of HPV16 for squamous cell carcinoma and of HPV18 for adenocarcinoma of the cervix compared to women with normal cytology in the Netherlands. Br J Cancer.

[24] Senba M, Kumatori A, Fujita S, Jutavijittum P, Yousukh A, Moriuchi T (2006). The prevalence of human papillomavirus genotypes in penile cancers from northern Thailand. J Med Virol.

[25] Bruni L, Barrionuevo-Rosas L, Albero G, Serrano B, Mena M, Gómez D, Muñoz J, Bosch FX, de Sanjosé S. ICO/IARC Information Centre on HPV and Cancer (HPV Information Centre). Human papillomavirus and related diseases in Thailand. Summary Report 17 June 2019.

[26] Wentzensen N, Nason M, Schiffman M, Dodd L, Hunt WC, Wheeler CM (2014). No evidence for synergy between human papillomavirus genotypes for the risk of high-grade squamous intraepithelial lesions in a large population-based study. J Infect Dis.

[27] Chaturvedi AK, Katki HA, Hildesheim A, Rodríguez AC, Quint W, Schiffman M (2011). Human papillomavirus infection with multiple types: pattern of coinfection and risk of cervical disease. J Infect Dis.

[28] Trottier H, Mahmud S, Costa MC, Sobrinho JP, Duarte-Franco E, Rohan TE (2006). Human papillomavirus infections with multiple types and risk of cervical neoplasia. Cancer Epidemiol Biomark Prev.

[29] Fernández-Nestosa MJ, Guimerà N, Sanchez DF, Cañete-Portillo S, Velazquez EF, Jenkins D (2017). Human papillomavirus (HPV) genotypes in Condylomas, intraepithelial Neoplasia, and invasive carcinoma of the penis using laser capture microdissection (LCM)-PCR: a study of 191 lesions in 43 patients. Am J Surg Pathol.

[30] Rantshabeng PS, Moyo S, Moraka NO, Ndlovu A, MacLeod IJ, Gaseitsiwe S (2017). Prevalence of oncogenic human papillomavirus genotypes in patients diagnosed with anogenital malignancies in Botswana. BMC Infect Dis.

[31] Freire MP, Pires D, Forjaz R, Sato S, Cotrim I, Stiepcich M (2014). Genital prevalence of HPV types and co-infection in men. Int Braz J Urol.

[32] Afonso LA, Carestiato FN, Ornellas AA, Ornellas P, Rocha WM, Cordeiro TI (2017). Human papillomavirus, Epstein-Barr virus, and methylation status of p16ink4a in penile cancer. J Med Virol.

[33] Senapati R, Nayak B, Kar SK, Dwibedi B (2017). HPV genotypes co-infections associated with cervical carcinoma: special focus on phylogenetically related and non-vaccine targeted genotypes. PLoS One.

[34] Hammer A, Rositch A, Qeadan F, Gravitt PE, Blaakaer J (2016). Age-specific prevalence of HPV16/18 genotypes in cervical cancer: a systematic review and meta-analysis. Int J Cancer.

[35] Hiller T, Poppelreuther S, Stubenrauch F, Iftner T (2006). Comparative analysis of 19 genital human papillomavirus types with regard to p53 degradation, immortalization, phylogeny, and epidemiologic risk classification. Cancer Epidemiol Biomark Prev.

[36] Vinokurova S, Wentzensen N, Kraus I, Klaes R, Driesch C, Melsheimer P (2008). Type-dependent integration frequency of human papillomavirus genomes in cervical lesions. Cancer Res.

[37] Takamoto D, Kawahara T, Kasuga J, Sasaki T, Yao M, Yumura Y (2018). The analysis of human papillomavirus DNA in penile cancer tissue by in situ hybridization. Oncol Lett.

[38] Ferreux E, Lont AP, Horenblas S, Gallee MPW, Raaphorst FM, von Knebel Doeberitz M (2003). Evidence for at least three alternative mechanisms targeting the p16INK4A/cyclin D/Rb pathway in penile carcinoma, one of which is mediated by high-risk human papillomavirus. J Pathol.

[39] Li J, Poi MJ (2011). Ming-Daw Tsai. The regulatory mechanisms of tumor suppressor P16INK4A and relevance to Cancer. Biochemistry..

[40] Halec G, Alemany L, Lloveras B, Schmitt M, Alejo M, Bosch FX (2014). Pathogenic role of the eight probably/possibly carcinogenic HPV types 26, 53, 66, 67, 68, 70, 73 and 82 in cervical cancer. J Pathol.

